# [^18^F]FDG-PET and [^18^F]MPPF-PET are brain biomarkers for the creatine transporter Slc6a8 loss of function mutation

**DOI:** 10.1038/s41598-025-92022-8

**Published:** 2025-03-01

**Authors:** Isabel Day, Mikayla Tamboline, Lindsay Lueptow, Irina Zhuravka, Taryn Diep, Ilona Tkachyova, Shili Xu, Andreas Schulze, Gerald S. Lipshutz

**Affiliations:** 1 Department of Surgery, David Geffen School of Medicine at UCLA, 757 Westwood Plaza, Room 8501G, Los Angeles, CA 90095-7054 USA; 2 Department of Molecular and Medical Pharmacology, David Geffen School of Medicine at UCLA, Los Angeles, CA USA; 3 Crump Institute for Molecular Imaging, David Geffen School of Medicine at UCLA, Los Angeles, CA USA; 4 Department of Psychology, David Geffen School of Medicine at UCLA, Los Angeles, CA USA; 5 Research Institute, The Hospital for Sick Children, University of Toronto, Toronto, ON M5G 1X8 Canada; 6 Jonsson Comprehensive Cancer Center, David Geffen School of Medicine at UCLA, Los Angeles, CA USA; 7 Departments of Paediatrics, The Hospital for Sick Children, University of Toronto, Toronto, ON M5G 1X8 Canada; 8 Departments of Biochemistry, The Hospital for Sick Children, University of Toronto, Toronto, ON M5G 1X8 Canada; 9 Department of Psychiatry and Biobehavioral Sciences, David Geffen School of Medicine at UCLA, Los Angeles, CA USA; 10 Molecular Biology Institute, David Geffen School of Medicine at UCLA, Los Angeles, CA USA; 11 Intellectual and Developmental Disabilities Research Center at UCLA, David Geffen School of Medicine at UCLA, Los Angeles, CA USA; 12 Semel Institute for Neuroscience, David Geffen School of Medicine at UCLA, Los Angeles, CA USA

**Keywords:** Creatine, SLC6A8 mutations, PET-CT, [^18^F]fluorodeoxyglucose, [^18^F]MPPF, CRT-1 transporter deficiency, Glucose, Serotonin, Development of the nervous system, Diseases of the nervous system, Neuroscience, Medical research

## Abstract

Pathogenic variants in the creatine transporter gene *SLC6A8*, reported to represent 2% of all intellectual disabilities in males, result in a spectrum of behavioral abnormalities including developmental delay, intellectual disability, and deficit in speech. While at present there are no effective treatments available, preclinical development and testing of gene therapy and other approaches to increase brain creatine are being actively pursued. In studying a mouse model of the disorder, [^18^F]fluorodeoxyglucose ([^18^F]FDG)-based positron emission tomography (PET)/computed tomography (CT) was performed to assess brain glucose metabolism in wild type and creatine transporter mutant mice (*Slc6a8*^*-/y*^). The findings demonstrate marked differences in glucose metabolism in the brains of wild type and *Slc6a8*^*-/y*^ mice. In conducting behavioral phenotyping studies, notable abnormalities in behavior in the murine model led to additional studies in serotonin-mediated activity. Serotonergic signaling differences were detected between wild type and *Slc6a8*^*-/y*^ mice using 4-(2′-methoxyphenyl)-1-[2′-(*N*-2″-pyridinyl)-*p*-[^18^F]fluorobenzamido]ethylpiperazine ([^18^F]MPPF). These data demonstrate that [^18^F]FDG-PET and [^18^F]-MPPF-PET may serve as appropriate and sensitive biomarkers that could be used to assess the efficacy of not only new approaches in treating mutations of the creatine transporter SLC6A8 and their effectiveness in normalizing brain metabolism but also in enhancing our understanding of the mechanism of brain dysfunction that occurs in this complex brain disorder.

## Introduction

The cerebral creatine deficiency syndromes, inborn errors of creatine metabolism, include two autosomal recessive creatine biosynthetic disorders [guanidinoacetate methyltransferase (GAMT) deficiency (MIM 601240) and L-arginine:glycine amidinotransferase (AGAT) deficiency (MIM 602360)] and the X-linked creatine transporter mutation (MIM 300036) affecting creatine uptake^1^. The hallmark of this family of disorders is the marked reduction or absence of creatine and phosphocreatine in the brain and the associated, predominantly neurological, disease ranging from mild to severe^2^. It has been suggested that these three conditions combined represent one of the most frequent metabolic disorders that consist of a primarily neurological phenotype^3^.

Solute carrier family 6 member A8 (*SLC6A8*) is the gene that encodes creatine transporter 1 (CRT-1), co-transporting the amino acid derivative creatine, (i.e., α-*N*-methyl-guanidino-acetic acid) with sodium and chloride across the cell membrane against a large concentration gradient^4,5^. Along with the kidney, SLC6A8 is highly expressed in tissues with high energy demand such as the brain, heart and skeletal muscle resulting in abundant intracellular creatine which plays an important role in energy storage and release within cells. As an intracellular energy reserve, phosphocreatine donates its phosphate group to ADP, rapidly regenerating ATP in the process^6^. Most cells rely on creatine uptake, not synthesis: in the brain, while endogenous synthesis may occur in some cells, this is limited and insufficient to meet the brain’s high energy demand. Furthermore, creatine pools must be replenished: about 1.7% of creatine degrades daily by nonenzymatic conversion into creatinine^3,6,7^. Thus, CRT-1 and creatine biosynthesis are relied upon heavily to maintain critical energy levels particularly in the brain.

With pathogenic variants of the creatine transporter, afflicted males are typically identified from workup for X-linked developmental disabilities. Creatine transporter deficiency (CTD) is the second most common disorder of X-linked neurodevelopmental conditions after Fragile X syndrome^8,9^ and is estimated to make up 2% of all cases of X-linked intellectual disability^8^. Patient presentation is usually with severely impaired language development (i.e. primarily expressive speech), developmental delay, intellectual disability, behavioral problems and seizures^2,10^; hypotonia is often present, and myopathy and hypotrophy of muscle can also be features. Autistic behavior may be a component, and microcephaly has been reported in some^11,12^. The areas of the human brain with the highest differentiated brain function–intellect, speech and behavior–are particularly affected^3^. There are also common constitutional symptoms and signs: gastrointestinal problems including constipation, feeding difficulties, failure to thrive, and attention deficit/hyperactivity. While most patients are male, about 50% of female carriers also have cognitive and behavioral issues^13^.

There is no effective treatment for the disorder^14^. As creatine does not cross the blood brain barrier without a transporter, the administration of massive doses of oral creatine is ineffective^15–17^. Gene-based therapies^18^ using rodent models deficient in functional CRT-1^19,20^ and demonstrating learning and memory deficits are in an investigational stage. Noninvasive methods to assess changes in brain function are limited. Magnetic resonance spectroscopy (MRS) can be used to measure biochemical changes in the brain, measuring levels of creatine and its precursors^21,22^. However, MRS has several limitations in monitoring potential treatment effects, including limited sensitivity to detect subtle changes in metabolite levels. MRS results also can change with age as metabolite levels in the brain can vary during development and thus reference values must be adjusted for age^23^; where more subtle changes may occur with therapy, differences may be difficult to detect and can vary based on voxel placement. Furthermore, MRS is technically complex requiring advanced equipment and a highly trained support staff^24^.

Because of the limitations of MRS, we sought to evaluate the use of positron emission tomography/computed tomography (PET/CT) for the study of functional loss of Slc6a8. PET is a functional imaging technique that can visualize metabolic activity by use of radiotracers taken up by tissues based on their metabolic rate^25^. [^18^F]labelled radiopharmaceuticals have been used to support clinical diagnoses and for monitoring patients with brain disorders. This includes [^18^F]FDG and amyloid tracers in Alzheimer’s disease, [^18^F]DOPA and [^18^F]FP-CIT in Parkinson’s, and [^18^F]fluorothymidine in brain tumors. In this study we used in vivo imaging with the FDA-approved glucose analog and radiotracer [^18^F]fluorodeoxyglucose ([^18^F]FDG) where activity concentrates in cells relying upon glucose for energy to evaluate if glucose metabolism in *Slc6a8*^*-/y*^ mice under resting conditions could be used as a biomarker, and to objectively assess glucose metabolism, to distinguish *Slc6a8*^*-/y*^ from wild type mice. Furthermore, we examined the expression of GLUT1 and 3, LKB1, CaMKKB, AGAT, and GAMT to gain a greater insight into difference in the brain metabolic activity of *Slc6a8*^*-/y*^ and wild type mice. In addition, based on unique findings in behavioral tests that were conducted as part of the studies herein, we also examined the serotonergic system with radiopharmaceutical probing with 4-(2′-methoxyphenyl)-1-[2′-(*N*-2″-pyridinyl)-*p*-[^18^F]fluorobenzamido]ethylpiperazine ([^18^F]MPPF), which has been used in clinical trials (completed: NCT01461083, NCT01461083, NCT01136213; enrolling: NCT06008704), and PET/CT imaging. Specifically, [18F]MPPF is a 5-HT1a receptor antagonist. Together, the studies herein of translational findings based on merging PET and CT imaging with radiopharmaceutical probing has led to a further understanding of the disorder correlating functional imaging from PET with the precise anatomical localization provided by CT.

## Methods

### Animal care and mouse procedures

Mice were housed in a vivarium at UCLA and kept according to the National Institutes of Health guidelines with temperature and humidity control. Experimental procedures were approved by and conducted according to the guidelines of the UCLA Chancellor’s Animal Care and Use Committee (ARC). Mice had *ad lib* access to standard chow (20% crude protein) (PicoLab Irradiated Rodent Diet 20, Catalog No. 5053, Lab Diet, Richmond, Indiana) and water and were maintained on a 12-h light–dark cycle. *Slc6a8*^*-/y*^ (Purchased from The Jackson Laboratory, Bar Harbor, ME, US, Stock 021,072) and littermate wild type mice (C57BL/6 background) were used for all studies^19^, housed on the same side of the mouse rack and on 1 of 3 rows. Mice were regularly monitored for pathogens in the UCLA A4 status specific pathogen free (SPF) colony. Genomic DNA was prepared from ear clip by standard methods and genotyping was performed by PCR amplification as described.

By random selection, adult mice 12–16 weeks of age were assigned to either control or experimental group. Male mice were represented throughout the study as this is an X-linked disorder. Blood sampling was obtained from the retro-orbital plexus under isoflurane anesthesia with plasma frozen immediately and stored at − 80 °C until analysis. Mice were euthanized by isoflurane overdose at the end of the live studies and before tissue collection.

### Analysis of metabolic profile from urine and plasma

Guanidinoacetic acid (GAA), as the immediate precursor of creatine undergoes methylation to produce creatine. Creatinine is produced by the spontaneous and nonenzymatic cyclization of creatine. The concentration of creatine, creatinine and guanidinoacetic acid was determined in samples using a 1260 liquid chromatography (LC) unit combined with triple-quad 6410B Mass Spectrometry (MS) (Agilent, Santa Clara, CA). Briefly, 10 µL of 1 mM internal standard (IS) epsilon amino caproic acid (EACA) was added to 10 µL of mouse plasma. For measuring creatinine, samples were deproteinized, dried down and reconstituted in 0.1% formate in H_2_O (Solution A) and used for analysis by LC–MS. For measuring creatine and guanidinoacetic acid (GAA), plasma was derivatized with 3N HCl-Butanol, heated for 15 min at 60 °C, then dried and reconstituted with 100 µL of solution A for LC–MS analysis. Separation was performed with an Agilent Poroshell 120 EC-C18 column with a mobile phase consistent of solution A and solution B (0.1% formate in acetonitrile and 0.005% trifluoracetic acid). Regarding underivatized samples, multiple reaction monitoring (MRM), 114-44 and 132-41 for creatinine and internal standard were used, respectively. For derivatized samples we used the MRM, 188-44, 188-69 and 174-101 for creatinine, GAA and internal standard, respectively. This was performed in a blinded manner.

### Analysis of metabolic profile from brain

#### Reagents

Tissue homogenization tubes, including VWR 2 mL × 2.8 mm Ceramic Hard Tissue Homogenizing Mix and VWR 2 mL × 1.4 mm Ceramic Soft Tissue Homogenizing Mix, were acquired from VWR (VWR International, Radnor, PA, USA). Formic acid (LC/MS grade) and methanol (HPLC grade) were sourced from Fisher Scientific (Ottawa, ON, Canada). Trichloroacetic acid (TCA) was obtained from VWR International (Radnor, PA). Buthanol·HCl (3 M) was supplied by Regis (Morton Grove, IL, USA). The chemicals used for calibrators and internal standards—guanidinoacetate, L-arginine, creatine, creatinine, ornithine-d6, arginine-d7, creatine-d3, and creatinine-d3—were purchased from Sigma-Aldrich Canada Co. (Oakville, ON, Canada). This was performed in a blinded manner.

#### Brain metabolites preparation

Thirty to 60 mg of brain was flash-frozen in 2 mL tubes with 1.4 mm ceramic beads. Each tube was filled with 1 mL of cold water and processed using the Omni Bead Raptor Elite. For these samples, the speed was set to 4.85 m/s for a single 20-s cycle. 300 µL of the tissue homogenates were mixed with 75 µL 30% TCA, vortexed, and centrifuged at 13,000 × rpm for 5 min to precipitate proteins. The cleared tissue homogenates were then transferred into Eppendorf tubes for storage at − 80 ℃ or processed immediately for metabolite extraction. For LC–MS/MS metabolite extraction, 10 µL of the cleared tissue homogenate was mixed with 10 µL of internal standard and 500 µL of methanol. The mixture was vortexed and centrifuged at 13,000 × rpm for 5 min, after which the supernatant was transferred into a clean glass test tube and loaded onto the Microvap (Organomation, Berlin, MA, USA) at 37 ℃ for evaporation of the excess solvent. The dry residue was dissolved in 100 µL buthanol·HCl (3 M), vortexed, and incubated at 60 °C for 30 min. Once cooled to room temperature, derivatized samples were again transferred onto the Microvap at 37 ℃ for evaporation of the excess solvent. The resulting dry residue was resuspended in 700 µL methanol and transferred into a 2 mL glass vial.

#### Liquid–chromatography tandem mass spectrometry (LC–MS/MS)

The LC–MS/MS method for analyzing creatine metabolites was adapted with slight modifications from Tran et al.^26^. The LC–MS/MS system consisted of an ExionLC AD UHPLC system coupled with QTRAP 6500plus (AB Sciex LLC, Framingham, MA). Metabolite separation was performed on a Kinetex C18 100 Å, 5 µm, 100 × 4.6 mm LC column (Phenomenex Inc., Torrance, CA, USA) using gradient binary elution at a flow rate of 0.7 mL/min and a temperature at 45 °C. The mobile phase consisted of solvent A (0.5 mmol/l ammonium formate, 0.1% (v/v) formic acid in water) and solvent B (0.5 mmol/l ammonium formate, 0.1% (v/v) formic acid in methanol). The elution gradient was performed as follows: 100% solvent A at 0 min; 100% solvent B at 5.0 min; 100% solvent B at 7.5 min; 100% solvent A at 7.55 min; 100% solvent A at 10 min. The injection volume was 1 µL. Mass spectrometry was conducted using the positive ionization and MRM scan mode. The optimized ion transitions were: creatine—188.2 → 90.0, creatinine—114.2 → 44.0, guanidinoacetate—174.2 → 101.1, creatine-d3—191.2 → 93.0, creatinine-d3—117.2 → 47.0, guanidinoacetate-d2—176.2 → 103.1. The ion source parameters were set at: TEM—600 °C, de-clustering potential—60.0, capillary voltage—5500 V, curtain gas—30, GS1—30, and GS2—20. Data processing and quantification were performed using Analyst 1.7.0 software (AB Sciex LLC, Framingham, MA, USA; https://sciex.com/products/software/analyst-software).

#### Calibrators and internal standard (IS) for LC–MS/MS

Calibrators stock solutions were prepared by weighing each compound individually using an analytical balance and dissolving each compound in water to final concentrations of 5 mM for creatine and creatinine, and 0.1 mM for guanidinoacetate. Working solutions of calibrators were prepared by serial dilution from the stock solutions, resulting in final concentrations of 500, 250, 100, 50, 25, 10, 5, 2.5, 0 µM for creatine and creatinine, and 10, 5, 2, 1, 0.5, 0.25, 0.1, 0.05, 0 µM for GAA. The internal standard (IS) mixture consisted of creatine-d3 and creatinine-d3 at concentration of 100 µM and guanidinoacetate-d2 at concentration of 10 µM, all dissolved in water. Calibrators and IS solutions were stored at − 20 °C until use. Analytes were quantified by calculating the signal intensity ratio of the analyte to its respective IS, and compared to external calibration using the signal intensity ratio of the calibrator to its respective IS. This was performed in a blinded manner.

### Blood glucose and plasma insulin determination

Blood collected retro-orbitally from fed anesthetized (between 1 and 2 pm) or fasted mice (6 h fasting with blood collected between 1 and 2 pm) was tested for whole blood glucose level using Precision Xtra test strips and glucometer, following the manufacturer’s instructions (Abbott Diabetes Care Inc., Alameda, CA, USA, 80050-65). Insulin was determined from 6 h fasted mice; plasma was collected from blood by centrifugation at 2000 g for 10 min at 4 °C and immediately frozen and stored at − 80 °C. Frozen plasma was sent on dry ice to IDEXX (West Sacramento, CA, USA) for insulin determination.

### Behavioral testing

Behavioral tests were performed during the light cycle (06:00–18:00) between the hours of 12:00 and 17:00. Mice were handled by the experimenters for 5 days prior to any behavioral testing to reduce handling stress during testing. All mice home cages were placed in the behavior room at least 1 h prior to testing to acclimate them to the environment. Experimenters were blinded to the treatment conditions. All behavioral testing included 9 mice wild type and 7 *Slc6a8*^*-/y*^ mice. All behavioral testing was performed by blinded scientists with group identification only released for analysis.

#### Open field and novel object recognition

The open field and novel object recognition (NOR) tests were performed as previously described^27–29^. Open field was performed to examine general locomotion and activity levels, exploration and curiosity, evidence of stereotypic or repetitive behaviors, and to examine for evidence of anxiety-related behavior. Novel object recognition was performed to examine recognition memory and cognitive function. The apparatus consisted of 40 cm × 40 cm grey acrylic plexiglass with 40 cm high walls (made in house). All testing was performed under low lighting (< 20 lx). On day one, mice were habituated to the empty open field and allowed to explore for 30 min. This habituation session was scored for overall locomotor activity, as well as anxiety-like behavior in the first 5 min (i.e., time and number of entries into the center of the open field). Twenty-four hours later, NOR training was performed with two identical objects placed in opposite corners of the field and mice were allowed to explore for 10 min. After a 30 min delay, one of the objects was replaced with a novel object and mice were again allowed to explore for 10 min for the testing phase. The discrimination index was calculated for the testing phase ([novel object exploration time—familiar object exploration time]/total exploration time). Any mice that did not reach the minimum exploration time of 20 s for both objects during either the training or testing phase were excluded from analysis. The objects, combinations of objects, and locations were used in a balanced manner to reduce potential bias. All sessions were recorded by overhead video and analyzed using AnyMaze software (Stoelting, version 7.3, Wood Dale, IL, USA; https://stoeltingco.com/Neuroscience/Anymaze/Any-maze-Video-Tracking/Any-maze-Software).

#### Two-bottle test for anhedonia

Two bottle testing for anhedonia was performed as described^30^ with slight modification. This behavioral test is to evaluate anhedonia, the reduced ability to experience pleasure. The study measures preference for a sweetened solution compared to water as mice typically prefer sweet tastes. As studies require temporary housing in individual cages to accurately measure fluid intake, we limited testing to a 2-h exposure protocol allowing for rehousing together after with minimum fighting. For each day of acclimation and testing, mice were placed in individual cages daily for 2 h. During acclimation days 1–5, two 50 mL French square glass bottles with rubber stoppers and metal lickits (VWR International, Radnor, PA, USA) were filled with 2% Sucrose (Millipore Sigma, Burlington, MA, USA) and placed on either side of the wire topper. On testing day 1, mice were given one bottle of water and one bottle of 2% sucrose. Total fluid intake was quite low, so on test days 2–4, mice were given one bottle of water and one bottle of 50%, 25%, and 5% Ensure across the 3 days, respectively. The concentration was titrated down from 50% Ensure in an attempt to overcome a potential ceiling effect on preference.

#### Cued and contextual fear conditioning

Fear conditioning^31^ is a test of associative learning primarily focused on hippocampus functioning with the involvement of the basolateral amygdala^31–33^. This testing examines fear learning and memory evaluating the ability to associate specific cues with experiences that are aversive, here a foot shock. Mice were placed into standard conditioning chambers with metal grid floor that reside within sound attenuating chambers (MedAssociates, Fairfax, VT, USA). Mice underwent pre-exposure to the context for 10 min to reduce baseline freezing. Twenty-four hours after the pre-exposure, subjects were placed back in the conditioning chamber for 3 min before the onset of the first foot shock unconditional stimulus (US; 0.75 mA, 2 s). Animals were exposed to three US’s in total, with an inter-trial interval of 1 min. After the third US, the mice were left in the conditioning chamber for another 60 s at which point they were retrieved from the conditioning chambers and then placed back in their home cages. Twenty-four hours later, the mice were returned to the same context as they were trained in and recorded for 8 min. No shocks were given during the test session. The next day mice were returned to the chambers and exposed to 30 presentations of the tone without the shock to extinguish freezing behavior. On the final day of testing, mice were again returned to the conditioning chamber and exposed to three tone-shock pairings with an inter-trial-interval of 1 min to induce reinstatement. The Video Freeze software version 1.0 (MedAssociates; https://med-associates.com/product/videofreeze-video-fear-conditioning-software/) was used to analyze the amount of movement to assess levels of freezing as a measure of fear of the context. A freezing threshold of 19 was set as the threshold to detect freezing based on prior hand scoring data obtained by the lab.

#### Light dark transition testing

Light dark transition testing was performed as previously described^34^. This behavioral test evaluates for evidence of anxiety-like and exploratory behavior. The light–dark box is used to measure anxiety-like behavior by utilizing the mouse’s natural preference for dark spaces. The light–dark box is a Plexiglas box (80 × 40 × 20 cm) divided into two compartments. The dark compartment consists of one-third the total box size with black walls, floor and ceiling. The light compartment consists of two-thirds the total box size and has white walls and floor and bright illumination (~ 500 lx). Between the two compartments is a 5 × 5 cm doorway. Each experimental session lasted for 10 min. The mouse was placed in the dark compartment for 5 min. The doorway between the two compartments was opened, and the behavior of the mouse was then recorded by videotape for 5 min. Each session was recorded and scored using AnyMaze software v. version 7.3 (https://stoeltingco.com/Neuroscience/Anymaze/Any-maze-Video-Tracking/Any-maze-Software). Videos were scored for the number of transitions between the light and dark compartments, initial latency to enter the dark compartment, and total time spent in the light compartment.

#### Marble burying and digging behavior

Marble burying^35^ and digging^36^ were performed as previously described. This form of behavior testing is employed in the evaluation for evidence of repetitive or compulsive behaviors and can be used to assess anxiety-related activity. It takes advantage of the natural instinct of mice to bury objects. The testing was performed in rat cages with double the bedding, which was flattened down into an even surface. Eighteen marbles were laid out in a grid pattern. Individual mice were placed in the center of the grid and left for 20 min. At the end of the session, the number of marbles covered at least 2/3 with bedding were counted. Additionally, digging behavior was scored for the first 5 min.

#### Nest building

Nestlet shredding^35,37^ was performed as previously described. This behavioral test evaluates the ability and motivation to building nests by shredding a nestlet. This is a natural behavior in mice. Mice were individually placed in clean cages containing 2 cotton nestlet squares for 2 h per day over 10 days. At the end of the 10 days, the nestlets were scored according to their appearance: (1) Nestlet mostly untouched; (2) Nestlet partially shredded with 50% or more remaining intact; (3) Nestlet mostly shredded but material dispersed around cage; (4) Nestlet mostly shredded (> 90%) and materially is gathered in a quarter of the cage, but the nest is flat; (5) Nestlet is mostly shredded and material built up into nest with crater.

### Micro-positron emission tomography (PET)/micro-computed tomography (CT) imaging

Mice were imaged as previously described^38^. [^18^F]MPPF was synthesized as described^39^ and [^18^F]FDG was purchased from PETNET Solutions (Culver City, CA, USA). In brief, mice were anesthetized in a chamber with 1.5% vaporized isoflurane, followed by tail vein injection in mice with [^18^F]FDG or [^18^F]MPPF under the unconscious condition to avoid restraint- and pain-induced stress effects on the brain metabolism and activities. There were no differences between dosing, volume, and molar activity between the mutant and wild type mice; they were kept consistent across all animals in the study. The scans of all animals, both the Slc6a8 and wild type, were performed in a random order and were completed within one half-life of ^18^F. Mice were transferred to a cage for a 60-min uptake period, during which they remained awake and freely moving. Mice were then anesthetized in a chamber with 1.5% isoflurane before undergoing micro-PET imaging with a 10-min static data acquisition. This was followed by microCT imaging immediately after. Images were acquired on a cross-calibrated GNEXT PET/CT scanner (Sofie Biosciences, Culver City, CA, USA). PET images were reconstructed using a 3D-ordered subset expectation maximization algorithm (24 subsets and 3 iterations), with random, attenuation, and decay correction. CT images were reconstructed using a Modified Feldkamp Algorithm. During analysis, PET data was corrected for radioactive decay and injected dose, and expressed as the percentage of injected dose per cubic centimeter (%ID/cc). Images were co-registered and PET/CT data were quantified using AMIDE software 1.0.4 (https://sourceforge.net/projects/amide/files/amide/1.0.4/)^40^. Quantification of [^18^F]FDG uptake in individual brain areas was performed using a mouse brain atlas as previously developed^41^.

### Quantitative real-time PCR

Tissue samples were collected from euthanized mice and total RNA was extracted from brains using the RNeasy Lipid Tissue Mini Kit (Qiagen, Hilden, DE, 74804) according to the manufacturer’s instructions. 1 ug of RNA was reverse transcribed using the High Capacity cDNA Reverse Transcription Kit (Applied Biosystems, Waltham, MA, USA, 4368814). Following cDNA generation, real-time PCR (qPCR) was performed using the SsoAdvanced Universal SYBR Green Supermix (BioRad, Hercules, CA, USA, 1725274) to quantify the expression of *Glut1* (F: TCAACACGGCCTTCACTG; R: CACGATGCTCAGATAGGACATC^42^), *Glut3* (F: TTCTGGTCGGAATGCTCTTC; R: AATGTCCTCGAAAGTCCTGC^42^), Liver Kinase B1 (*Lkb1*) (F: TTGGGCCTTTTCTCCGAGG; R: CAGGTCCCCCATCAGGTACT^43^), *Cammkb* (F: CCTACGGCAAACCTGTGGACAT: R: GCCTTGATCTGCTGGTACAGCT [Origene, Rockville, MD, USA, MP201595]), *Gam*t (F: TGCCTGACGGTCACTTTGATGG; R: GACGTGAGGTTGCAGTAGGTGA [Origene, MP205827]), *Agat* (F: TCACGCTTCTTTGAGTACCG; R: TCAGTCGTCACGAACTTTCC^44^), and *β-actin* (F: CTAAGGCCAACCGTGAAAAG; R: ACCAGAGGCATACAGGGACA) genes. Melting temperatures for primer sets were 55 °C, 55 °C, 58 °C, 59 °C, 59 °C, 55 °C, and 56 °C respectively. Quantification was determined via threshold cycle (C_T_) values using the MyiQ2 Two Color Real-Time PCR Detection System (BioRad, 170-9790). The qPCR protocol for *Glut1*, *Glut*3, and *Lkb1* was performed as follows: DNA denaturation for 3 min at 95 °C, primer annealing for 10 s at 95 °C, then primer extension for 25 s at 56 °C. The qPCR protocol for *Cammkb*, *Gam*t, and *Agat* was performed as follows: activation at for 2 min at 50 °C, DNA denaturation for 10 min at 95 °C, primer annealing for 15 s at 95 °C, then primer extension for 60 s at 60 °C. Primer annealing and extension steps were repeated for up to 40 cycles for all protocols. All target gene expression was normalized to endogenous *β-actin* and relative fold enrichment of the mutant *Slc6a8* group was calculated using the 2^-ΔΔCt^ in comparison to the wild type group.

### Statistical analysis

Collected data was analyzed with a statistical software package (GraphPad Prism 10 Software, San Diego, CA; https://www.graphpad.com/). Results were expressed as mean ± standard deviation (SD). *P* values were determined using two-way ANOVA, mixed-effects analysis with the Geisser-Greenhouse correction, or unpaired T-test with Welch’s correction when applicable. All data is presented as means and error bars represent SD. *p* < 0.05 was considered significant.

*Behavioral testing*: Open Field: Welch’s t test (total distance traveled, center entries, center time), mixed-effects analysis (distance traveled over time, mean speed over time); Light–dark: Welch’s t test; Novel Object Recognition: Welch’s t test; Fear Conditioning: mixed-effects analysis, Welch’s t test (total freezing); Ensure Preference: two-way ANOVA (50% Ensure, 25% Ensure, 5% Ensure), Welch’s t test (50% Ensure preference score, 25% Ensure preference score, 5% Ensure preference score); Marble Burying: Welch’s t test; Nest Building: Welch’s t test. All data is presented as mean ± standard deviation (SD).

*PET/CT imaging*: Welch’s t test.

*Brain/plasma creatine, creatinine, and GAA testing*: Welch’s t test.

*Glucose/insulin testing*: Welch’s t test.

## Results

The authors have made every effort to conduct and report this study in accordance with the ARRIVE Essential 10 guidelines (The ARRIVE guidelines 2.0, National Centre for the Replacement Refinement & Reduction of Animals in Research, July 2020). All data points were included in the analyses (i.e., no data points were excluded as this was established *a priori*).

### Plasma creatine and brain creatine are markedly reduced in a *Slc6a8*^-/y^ murine model

**Fig. 1:**
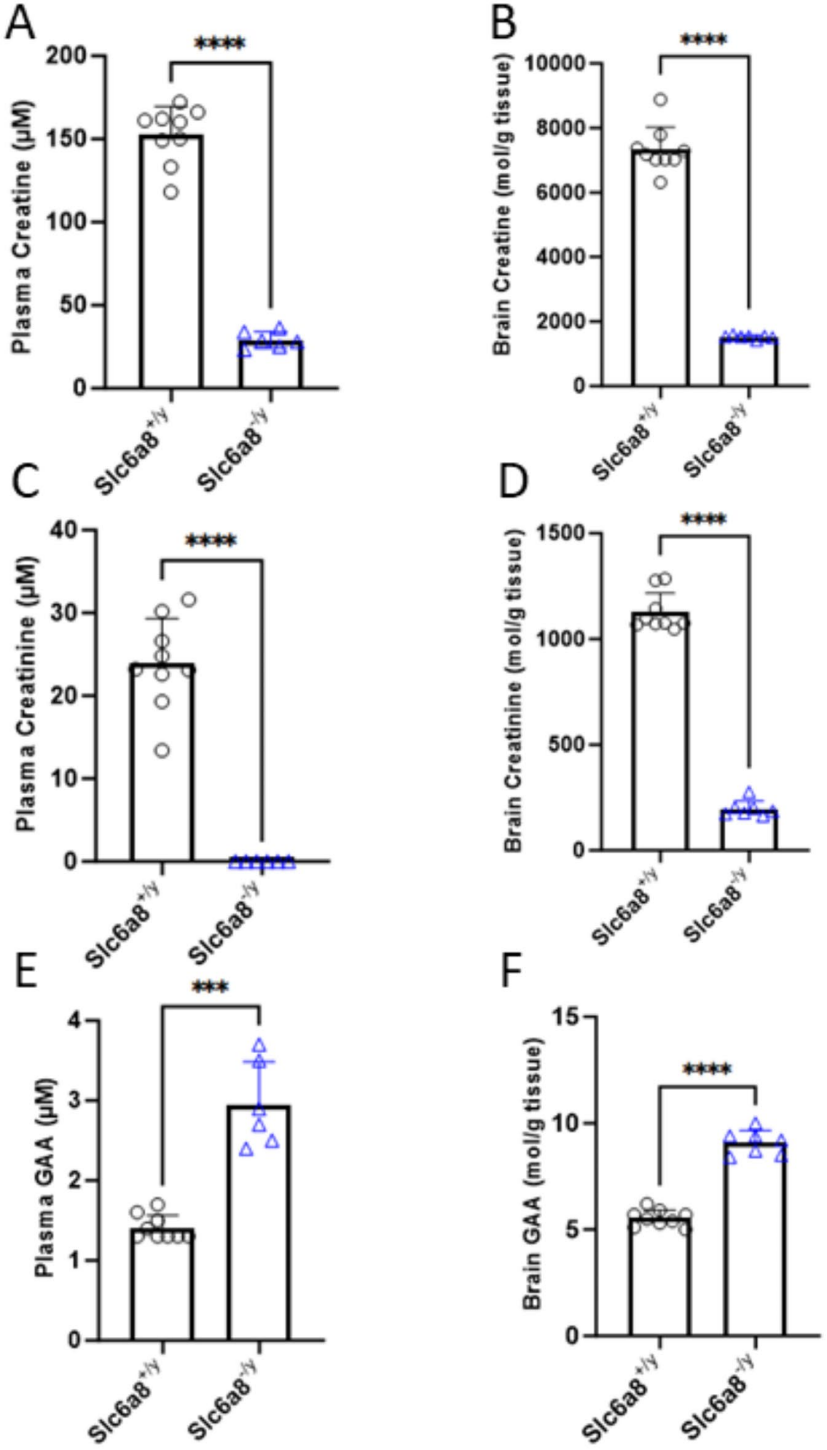
Plasma and brain creatine and creatinine levels are markedly reduced in *Slc6a8*^-/y^ mice while guanidinoacetic acid is mildly increased. (**A**) Plasma creatine, (**B**) brain creatine, (**C**) plasma creatinine, (**D**) brain creatinine, (**E**) plasma guanidinoacetic acid, and (**F**) brain guandinoacetic acid. All data is presented as mean + standard deviation. *P* values between 0.0001 and 0.001 are shown with three (***), and *P* values less than 0.0001 are shown with four (****). (GAA, guanidinoacetic acid)

Adult *Slc6a8*^+*/y*^ (i.e., wild type [WT]) and *Slc6a8*^*-/y*^ were studied. Plasma was collected, mice were euthanized, and brains were removed with the frontal lobe quantified for amount of creatine, creatinine and GAA present. Plasma creatine concentration was 152.30 ± 17.20 µmoles/l (n = 9) in WT mice while in *Slc6a8*^*-/y*^ mice plasma creatine was markedly reduced at 29.12 ± 4.98 µm/l (n = 7) (*p* < 0.0001), a reduction of ~ 81% (Fig. 1A). The decline in brain tissue creatine was similarly reduced: in WT mice, brain creatine was 7318.00 ± 703.2 mol/g brain tissue (n = 9) while in *Slc6a8*^*-/y*^ mice creatine was 1524.00 ± 55.14 mol/g brain tissue (n = 7) (*p* < 0.0001), a reduction of 79% and suggesting that in this mouse model the brain’s endogenous synthesis capability may produce ~ 20% of normal creatine levels (Fig. 1B).

Creatinine, the non-enzymatic metabolic product of creatine and creatine phosphate, was also reduced. Plasma creatinine (Fig. 1C) was undetectable in *Slc6a8*^*-/y*^ mice (n = 7) while in WT mice it was 23.87 ± 5.49 µm/l (n = 9) (*p* < 0.0001). Creatinine levels in the brain, likely due to the endogenous synthesis of creatine, were 17.5% of WT (WT: 1126.00 ± 91.50 [n = 9] mol/g brain tissue vs. *Slc6a8*^*-/y*^: 197.20 ± 37.75 mol/g brain tissue [n = 7] (*p* < 0.0001) (Fig. 1D), similar to the percent value of brain tissue creatine.

In the plasma of *Slc6a8*^*-/y*^ mice (n = 7), GAA is mildly elevated compared to WT mice (n = 9) (Fig. 1E) (2.95 ± 0.54 µM vs. 1.41 ± 0.15 µM; *p* < 0.0001). Similarly, GAA is mildly elevated in the brain in *Slc6a8*^*-/y*^ mice (Fig. 1F) (9.09 ± 0.58 µM vs. 5.53 ± 0.38 µM; *p* < 0.0001).

### Anxiety-like behavior is a feature of *Slc6a8*^-/y^ mice

**Fig. 2:**
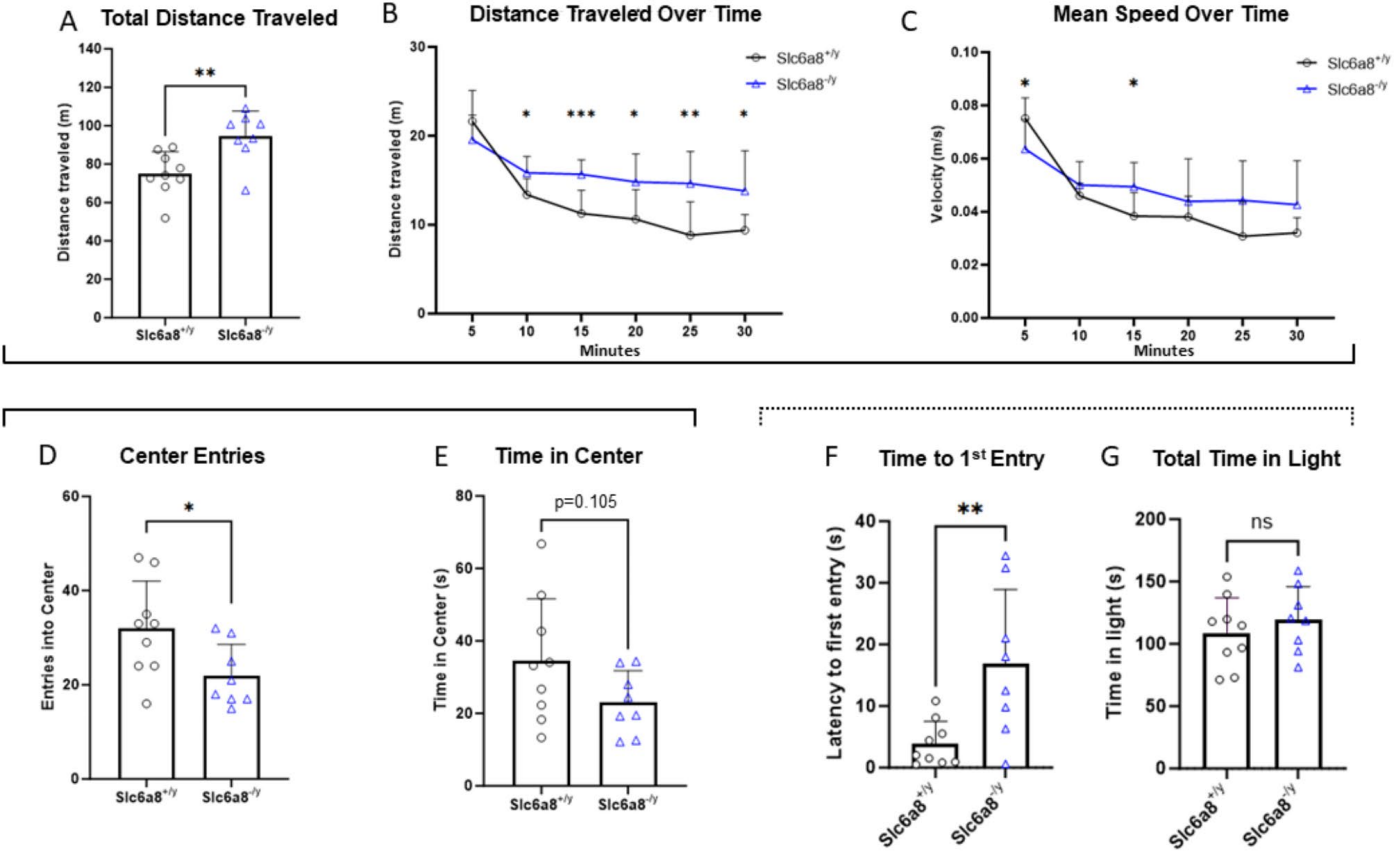
Open field and light dark transition testing revealed an anxiety behavioral phenotype in creatine transporter mutated mice. Open field testing results are depicted in A-E (solid bracket) with (**A**) total distance traveled in meters, (**B**) distance traveled over time in meters, (**C**) velocity over time in m/s, (**D**) number of entries to the center and (**E**) time in center of the open field. Light dark transition results (dotted bracket) are presented in (**F**) time to first entry and (**G**) total time in light. Data are presented as mean + standard deviation. *P* values of ≤ 0.05 are shown with one (*) asterisk, ≤ 0.01 are shown with two (**), and between 0.0001 and 0.001 are shown with three (***).

Open field testing (Fig. 2, black solid line bracket) was used to assess locomotion, anxiety, and exploratory behavior of *Slc6a8*^*-/y*^ mice compared to WT mice. Examining total locomotive behavior, *Slc6a8*^*-/y*^ mice had an increase in both the total distance traveled (Fig. 2A) (WT: 75.13 ± 11.28 m vs. *Slc6a8*^*-/y*^: 94.38 ± 13.17 m, *p* = 0.0062) and distance traveled across the 30 min session as compared to WT mice (Fig. 2B; Time × Genotype interaction, F (5, 75) = 5.834, *p* = 0.0001; Main effect of genotype, F (1,15) = 10.55, *p* = 0.0054; main effect of time, F (3.385, 50.78) = 33.75, *p* < 0.0001). While the mean speed of *Slc6a8*^*-/y*^ mice was decreased compared to WT during the first 5 min (WT: 0.075 ± 0.006 m/s; *Slc6a8*^*-/y*^: 0.063 ± 0.075 m/s; *p* = 0.0317), *Slc6a8*^*-/y*^ speed remained more stable across the session, and the WT mice speed decreased (Fig. 2C; main effect of time, F (3.264, 45.69), *p* < 0.0001; Time x Genotype interaction, F (5, 70) = 5.217, *p* = 0.0004). Taken together, this data suggests a hyperlocomotive phenotype in the mutant mice and/or an inability to habituate to a novel environment over time.

Mice typically prefer to stay near the walls of an open space, a defensive strategy known as thigmotaxis^45^. Thigmotaxis gradually decreases over the first 5 min of an open field session, with mice spending progressively more time in the center, and aberrations in this behavior can indicate an anxiety-like phenotype. *Slc6a8*^*-/y*^ mice had a decreased number of center entries (Fig. 2D; WT: 31.89 ± 10.15 center entries/5 min vs. *Slc6a8*^*-/y*^: 22.00 ± 6.61 center entries/5 min; *p* = 0.031) and a trend towards decreased time in the center (Fig. 2E; WT: 34.44 ± 17.18 s in center/5 min vs. *Slc6a8*^*-/y*^: 23.06 ± 8.69 s in center/5 min; *p* = 0.105) as compared to WT mice, indicating an anxiety-like phenotype.

To further probe this potential anxiety-like phenotype, all mice were exposed to the light/dark transition test (Fig. 2, dotted bracket), which assesses a mouse’s willingness to explore a bright, more aversive open field versus remaining in a smaller, dark compartment. This test measures the balance between the innate spontaneous exploratory behavior of a mouse in a new environment versus the aversive, brightly lit areas, where a longer latency to entry and/or less time in the light side indicates an anxiety-like phenotype is present. *Slc6a8*^*-/y*^ mice had a prolonged latency to first entry into the light compartment as compared to WT (Fig. 2F; WT 3.8 ± 3.7 s (n = 9) vs. *Slc6a8*^*-/y*^ 16.9 ± 12.0 s (n = 8); *p* = 0.018). However, total time (cumulative of all visits over 5 min in the bright lit area) (Fig. 2G) was similar between groups: WT 108.9 ± 28.1 s, *Slc6a8*^*-/y*^ 119.5 ± 26.4 s (*p* = 0.435). Together with the decrease in center time, the *Slc6a8*^*-/y*^ mice demonstrate an anxiety-like phenotype.

### Alteration in associative learning and memory are features of *Slc6a8*^-/y^ mice

**Fig. 3:**
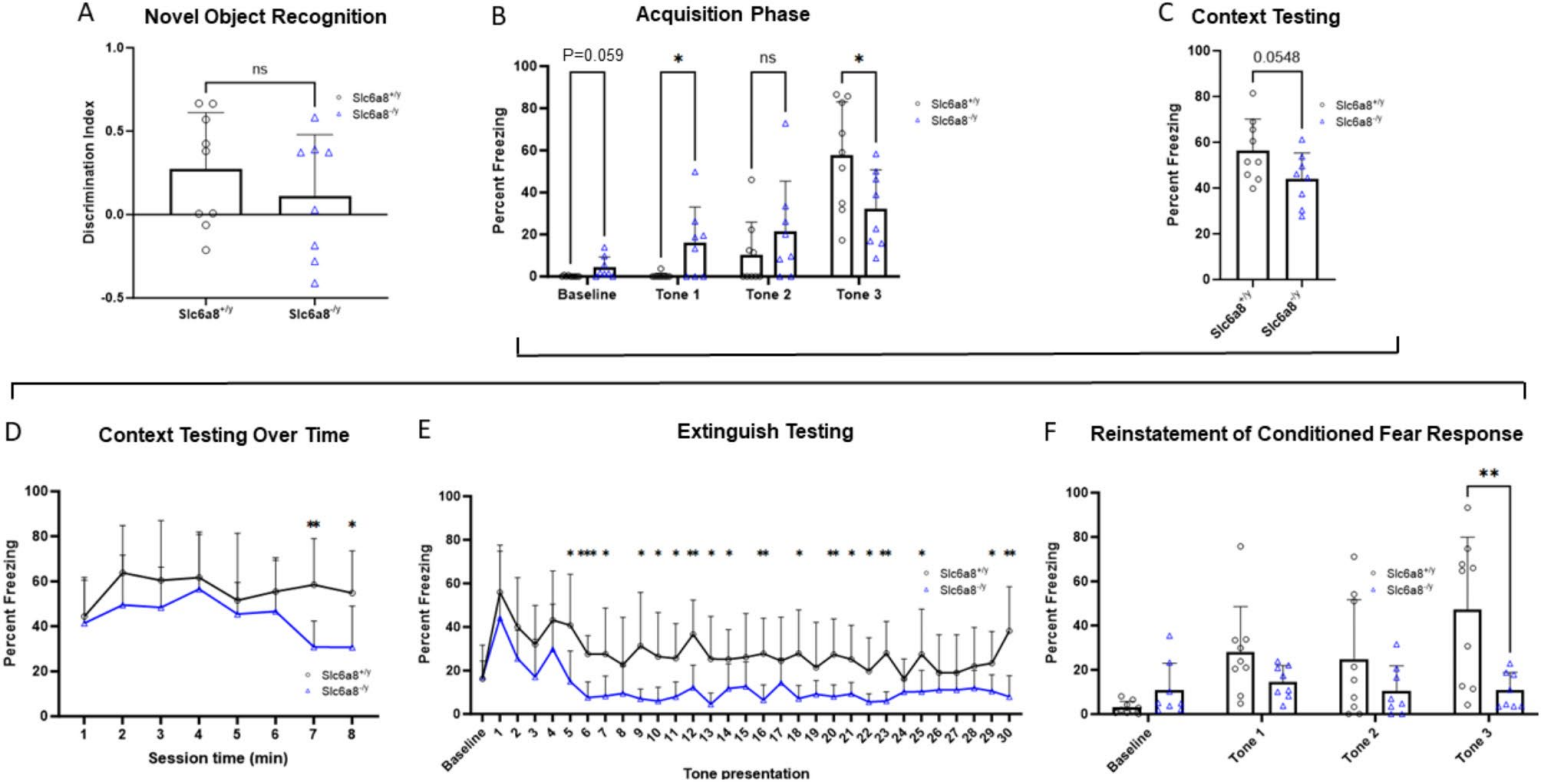
While novel object recognition is similar to wild type mice, *Slc6a8*^*-/y*^ mice show abnormalities in conditioned fear testing. (**A**) Novel object recognition testing demonstrates no statistically significant difference between *Slc6a8*^*-/y*^ and WT mice. Conditioned/contextual fear testing was performed with (**B**) acquisition phase, (**C**) context testing, (**D**) context testing over time, (**E**) extinguish testing, and (**F**) testing for reinstatement of conditioned fear response. All data is presented as mean + SD. WT = wild type. *P* values of ≤ 0.05 are shown with one (*) asterisk, ≤ 0.01 are shown with two (**), and between 0.0001 and 0.001 are shown with three (***).

Novel object recognition testing assesses learning and recognition memory based on the natural tendency of rodents to explore novelty. This form of recognition memory is dependent on the entorhinal and perirhinal cortex^46^. The Discrimination Index (DI), while reduced in *Slc6a8*^*-/y*^ mice, was not statistically significant in its difference from wild type mice (WT: 0.272 ± 0.340; *Slc6a8*^*-/y*^: 0.109 ± 0.370; *p* = 0.363) (Fig. 3A). In one sample t-tests vs zero, which would indicate no recognition memory, WT mice demonstrated recognition memory (*p* = 0.0432) but mutant mice did not (*p* = 0.432), potentially indicating a cognitive impairment.

Fear conditioning testing (Fig. 3, black bracket) is used to study associative learning and memory. It is based on classical conditioning principles where a neutral stimulus, here a sound (i.e., tone) and/or the environment becomes associated with an aversive event, such as a mild foot shock. In the acquisition phase (Fig. 3B), mutant mice had a slightly higher baseline freezing to the novel environment (*p* = 0.059; WT 0.138 ± 0.274 vs. *Slc6a8*^*-/y*^ 4.213 ± 5.125). During the presentation pairing of the tone and foot shock, both WT and mutant mice demonstrated increases in freezing to the tone across the session, an indication of learning (Main effect of time, F (2.214, 33.21) = 30.07; *p* < 0.0001). However, by the third and final tone presentation, mutant mice had slightly reduced freezing as compared to the WT mice (*p* = 0.033; Time x Genotype interaction, F(3, 45) = 7.107; *p* = 0.0005).

For context testing, the mouse is returned to the original training environment for 8 min and percent freezing is measured. Mutant mice had a trend towards lower *total* freezing compared to WT mice (Fig. 3C; freezing, WT: 56.62 ± 13.61% vs 43.84 ± 11.48%, *p* = 0.0548). Across the 8-min session, mutant mice had a trend towards lower freezing over time (Main effect of genotype, F (1,15) = 4.204; *p* = 0.0582) (Fig. 3D). Though not quite statistically significant, this data indicates a potential impairment of contextual fear memory in the mutant mice.

Ability to extinguish freezing behavior was also assessed (Fig. 3E). The mice were exposed to the conditioned stimulus (i.e., tone) multiple times without the foot shock. In response to the presentation of repeated tones, both the WT and the *Slc6a8*^*-/y*^ mice showed a marked reduction in percent freezing (i.e., Tone 1 vs. Tone 30; Main effect of time, F (7.187, 107.8) = 5.225; *p* < 0.0001), indicating that the conditioned fear response was decreased over time. However, mutant mice extinguished more quickly and to a greater extent (Main effect of genotype, F (1,15) = 20.35; *P* = 0.0004). Furthermore, mutant mice were resistant to reinstatement (Fig. 3F), referring to the recovery of the conditioned fear response (i.e., freezing) after extinction. Mice were placed back in the same environment and exposed to 3 additional tone-shock pairings. By the third pairing, WT mice had significantly increased freezing as compared to the *Slc6a8*^*-/y*^ mice (*p* = 0.0099). In aggregate the data suggest mild impairment in both cued and contextual fear conditioning in *Slc6a8*^*-/y*^ mice, with lower freezing when returned to the training context, as well as faster extinction and impaired reinstatement to the cue. The implication is that there appears to be some dysfunction of the amygdala encoding the association of the tone and the foot shock with expression of the fear response (i.e. freezing)^47^, along with dysfunction of the hippocampus in contextual memory and basolateral amygdala in processing and integrating sensory information and its relationship to fear. In addition, alterations in the serotonergic system are known to impair fear behavior^48^, and abnormalities in serotonergic signaling may play a greater role in behavioral abnormalities in *Slc6a8*^*-/y*^ mice than previously appreciated.

### Ensure preference testing suggest *Slc6a8*^-/y^ mice may have a nutritional deficiency

The Ensure nutritional supplement preference test assesses taste preference and pleasure-related responses. It is high in calories and palatable and can be used to evaluate the reward system in mice. While traditionally the test is performed with 1–2% sucrose, we did not observe high enough drinking volumes over 2 h to draw any meaningful conclusions (data not shown). Thus, we instead used the more palatable and highly rewarding Ensure, diluted from 5 to 50% to match the sugar content traditionally used with sucrose testing (e.g., 2% or less). Here mice are given a two-bottle choice between water and Ensure and the amount of liquid consumed by singly housed mice over a two-hour period was measured.

**Fig. 4:**
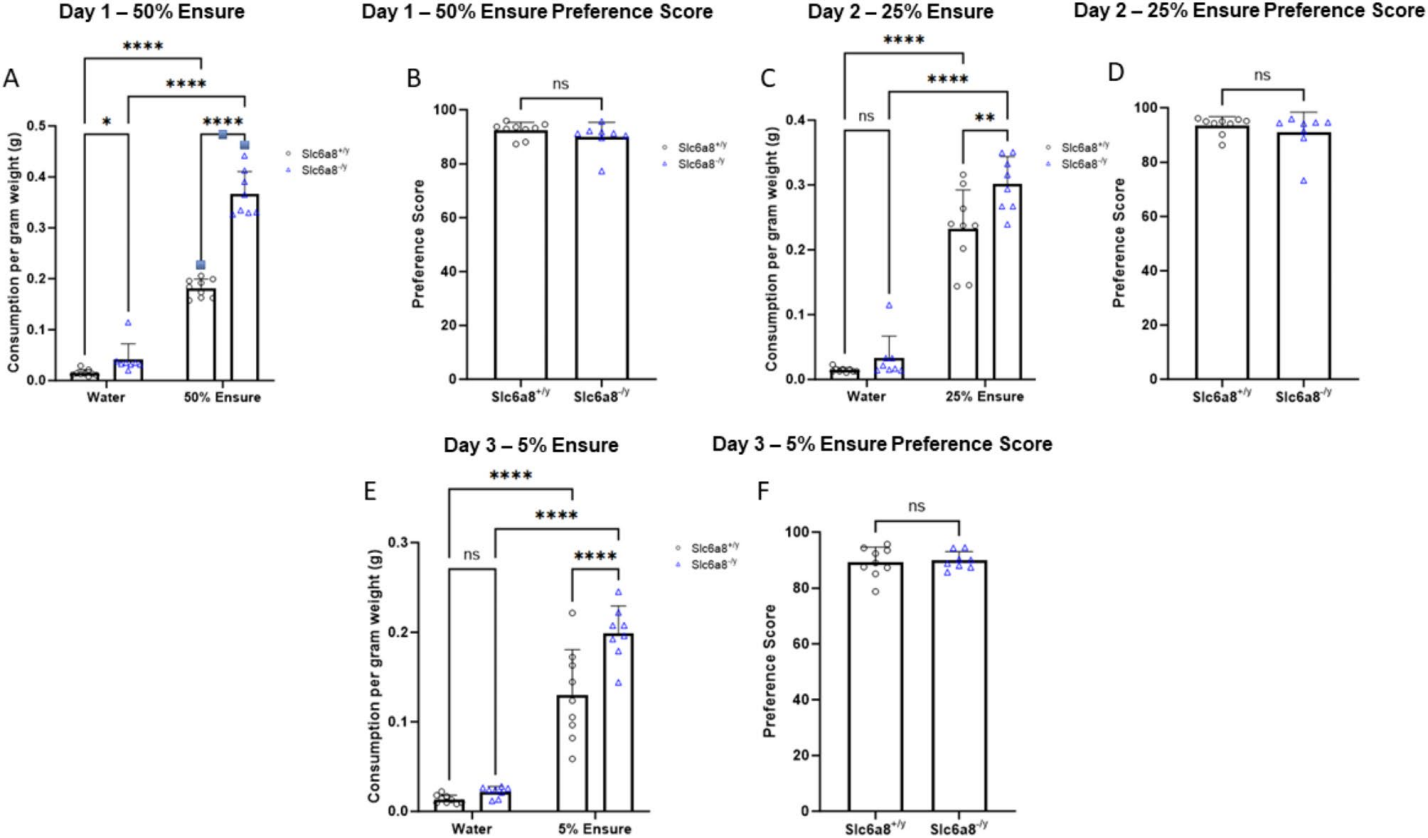
Testing for preference demonstrates higher consumption of both water and Ensure in *Slc6a8*^*-/y*^ mice, and greater intake of Ensure over water in *Slc6a8*^*-/y*^ mice. Day 1 testing with 50% Ensure for (**A**) consumption and (**B**) preference. Day 2 testing with 25% Ensure for (**C**) consumption and (**D**) preference. Day 3 testing with 5% Ensure for (**E**) consumption and (**F**) preference. Data is presented as mean + standard deviation. *P* values of ≤ 0.05 are shown with one (*) asterisk, ≤ 0.01 are shown with two (**), between 0.0001 and 0.001 are shown with three (***), and *P* values less than 0.0001 are shown with four (****).

Day 1 testing with 50% Ensure demonstrated that *Slc6a8*^*-/y*^ mice consumed more of each liquid compared to WT mice (Fig. 4A). With regards to water, WT mice consumed 0.0149 ± 0.006 ml per gram weight while mutant mice consumed 0.0423 ± 0.0296 ml per gram weight (*p* = 0.049); with 50% Ensure, WT mice consumed 0.1813 ± 0.0179 ml per gram weight while mutant mice consumed 0.3665 ± 0.0442 ml per gram weight (*p* < 0.0001). The amount of Ensure consumed by both groups was significantly higher than water (*p* < 0.0001). However, the preference score [amount Ensure consumed ÷ (water + Ensure consumed) *100] for Ensure by WT and creatine transporter mutant mice was similar (WT: 92.47% ± 2.89; *Slc6a8*^*-/y*^: 89.96% ± 5.40) and not statistically different (*p* = 0.266) (Fig. 4B). Similar findings were found for Day 2 with 25% Ensure (Fig. 4C) (water: WT mice consumed 0.0146 ± 0.003 ml per gram weight while mutant mice consumed 0.0328 ± 0.0341 ml per gram weight [*p* = 0.363]; Ensure: WT mice consumed 0.2320 ± 0.0605 ml per gram weight while mutant mice consumed 0.3021 ± 0.0417 ml per gram weight [*p* = 0.001]) with no difference in preference (Fig. 4D) (WT: 93.55% ± 3.24; *Slc6a8*^*-/y*^: 90.94% ± 7.47; *p* = 0.383) and Day 3 with 5% Ensure (Fig. 4E) were similar (water: WT mice consumed 0.0135 ± 0.0464 ml per gram weight while mutant mice consumed 0.0218 ± 0.0061 ml per gram weight [*p* = 0.576]; Ensure: WT mice consumed 0.1296 ± 0.0508 ml per gram weight while mutant mice consumed 0.1993 ± 0.0299 ml per gram weight [*p* < 0.0001]) with again no difference in preference between genotypes (Fig. 4F) (WT: 89.32% ± 5.37; *Slc6a8*^*-/y*^: 89.95% ± 3.14; *p* = 0.7678). These data suggest that the *Slc6a8*^*-/y*^ mice could have a nutritional deficiency (or may need greater glucose intake) which results in increased Ensure consumption. While the preference score between the WT and mutants is the same, a ceiling effect could have been reached.

### Alteration in behavior of *Slc6a8*^-/y^ mice are consistent with alteration in levels of serotonergic activity

**Fig. 5:**
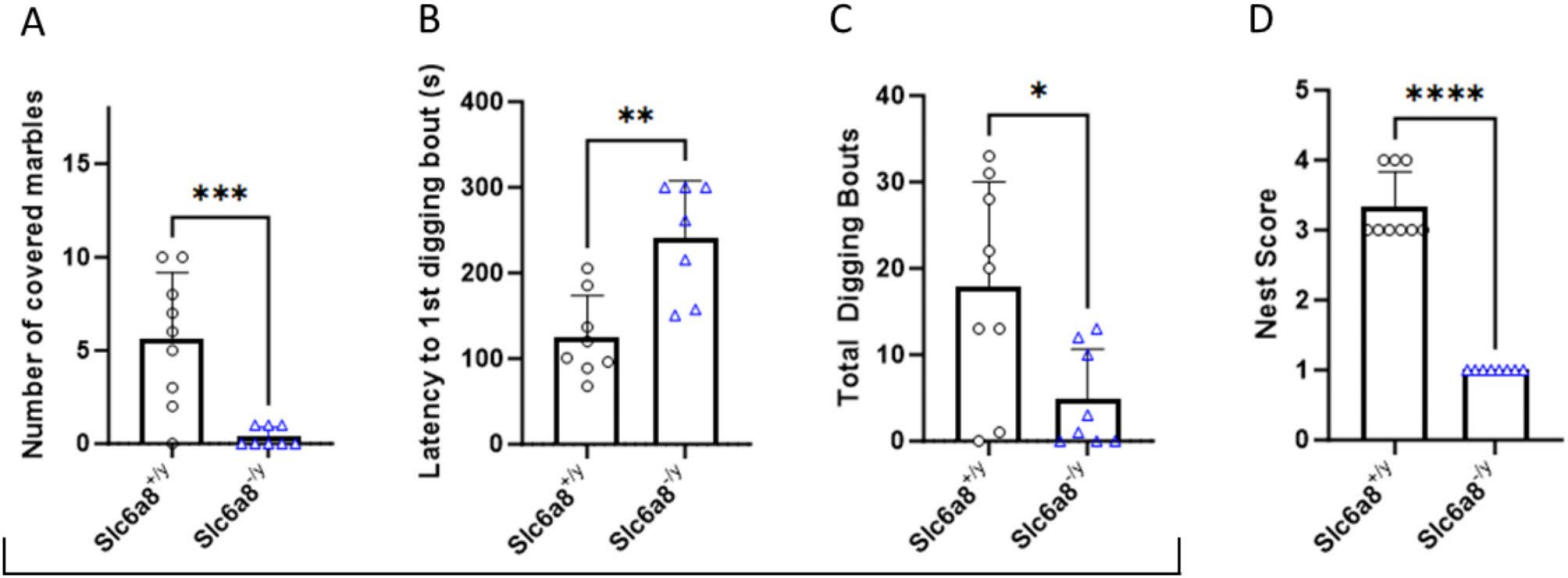
Marked abnormalities in marble burying, digging and nest building are present in creatine transporter mutated mice. Marble burying (solid bracket) in (**A**) number of marbles buried, (**B**) latency to first episode of digging, and (**C**) total time digging. Creation of a nest was scored and is depicted in (**D**). Data is presented as mean + standard deviation. *P* values of ≤ 0.05 are shown with one (*) asterisk, ≤ 0.01 are shown with two (**), between 0.0001 and 0.001 are shown with three (***), and *P* values less than 0.0001 are shown with four (****).

The marble burying test (Fig. 5, black bracket) can be used to assess repetitive and compulsive-like tendencies, taking advantage of the natural digging behavior of mice. Excessive burying can be seen as a form of a repetitive or compulsive behavior. *Slc6a8*^*-/y*^ mice do not show this behavior but instead demonstrate markedly reduced activity. Creatine transporter mutated mice bury almost no marbles over a 20-min time period (Fig. 5A): wild type mice bury 5.67 ± 3.50 marbles (n = 9) while mutant mice bury 0.38 ± 0.52 marbles (n = 8) over the same time period (*p* = 0.0018). There is a prolonged latency (92% longer compared to WT) to the first digging episode (Fig. 5B) (WT: 125.20 ± 48.29 s; *Slc6a8*^*-/y*^: 240.80 ± 66.63 s; *p* = 0.0030) and a marked reduction (~ 73%) of digging bouts (Fig. 5C) (WT: 17.89 ± 12.13 s; *Slc6a8*^*-/y*^: 4.88 ± 5.77 s over the 5-min time period; *p* = 0.0143). Furthermore, nest building, a natural behavior for mice associated with well-being, is markedly reduced in mutant mice (Fig. 5D): nest score in WT mice is 3.33 ± 0.50 shredded vs *Slc6a8*^*-/y*^ where it is 1.00 ± 0.00 shredded; *p* < 0.0001). Together this clustering of findings suggests a general reduction in motivational, exploratory and self-care behaviors. Previous research has demonstrated a strong role for the serotonergic system in modulation of these behaviors, with enhanced serotonergic tone correlated with decreased performance in these tests (e.g., decreased marble burying, decreased digging behavior, and decreased nesting behavior)^49,50^.

### [^18^F]FDG-PET/CT imaging demonstrates increased glucose metabolic activity in the brain, heart and skeletal muscle of Slc6a8^-/y^ mice with reduced blood glucose and insulin levels

**Fig. 6:**
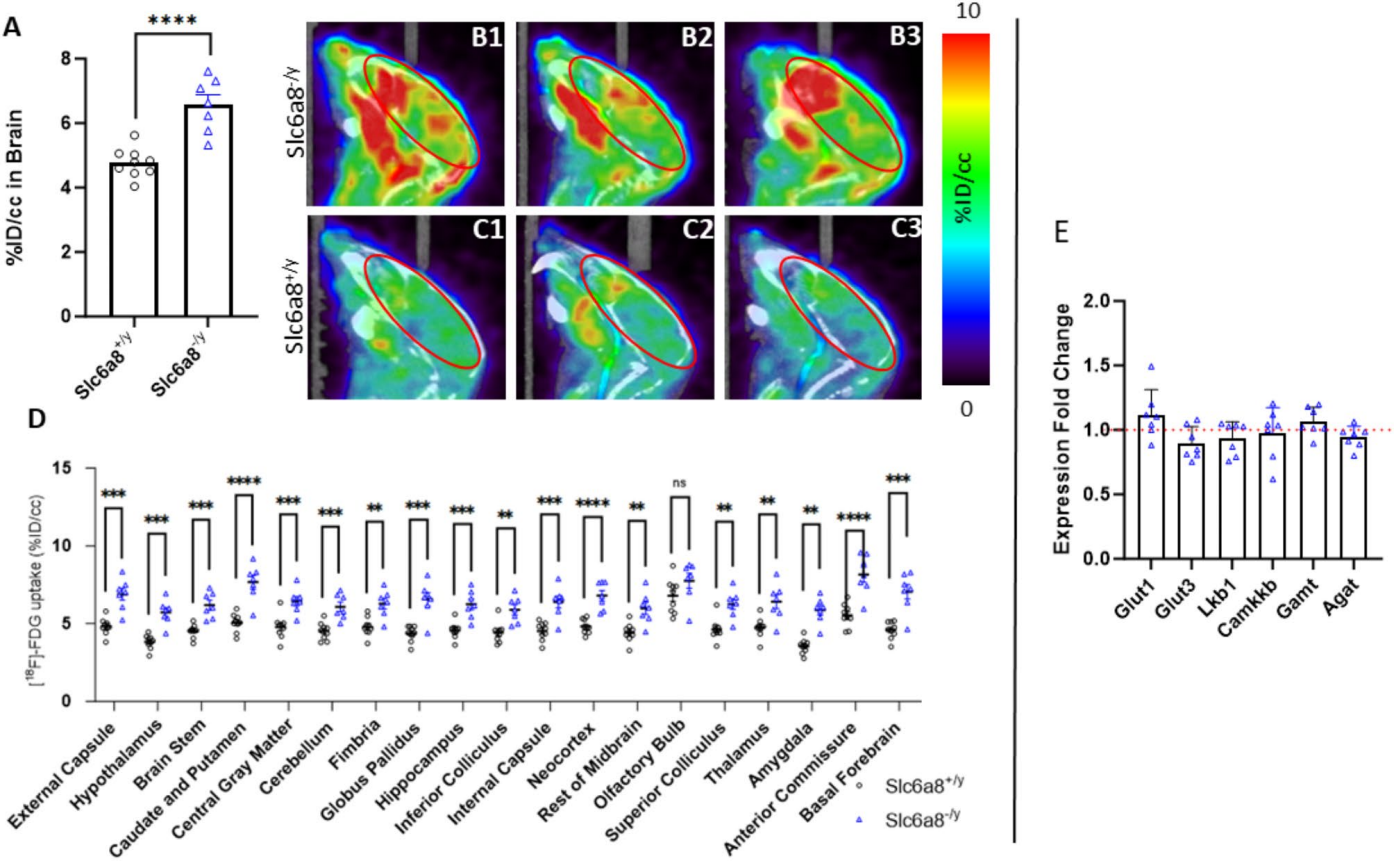
Glucose metabolism is increased throughout the brain in creatine transporter mutated mice. Increased glucose metabolism in the brain occurs in *Slc6a8*^*-/y*^ mice (**A**, representative mice in **B1**, **B2**, **B3**) as compared to WT (representative images in **C1**, **C2**, **C3**). Individual brain regions were evaluated (**D**) and were statistically significant in their differences in all brain regions except the olfactory bulbs. Genes related to glucose uptake, energy charge, and creatine biosynthesis (**E**), in *Slc6a8*^*-/y*^ mice were compared to WT mice for differences (WT level normalized to 1.0 and represented by the red dotted line). Data is presented as mean + standard deviation. FDG = fluorodeoxyglucose, %ID/cc = percent injected dose per cubic centimeter. *P* values of ≤ 0.01 are shown with two (**), between 0.0001 and 0.001 are shown with three (***), and *P* values less than 0.0001 are shown with four (****).

Mice were intravenously administered [^18^F]FDG and 1 h later underwent PET/CT imaging (WT: n = 9; *Slc6a8*^*-/y*^, n = 7) (Fig. 6). *Slc6a8*^*-/y*^ mice with mutated creatine transporter demonstrated increased activity as concluded by the percentage of injected dose per cubic centimeter (%ID/cc) in the brain of *Slc6a8*^*-/y*^ compared to WT mice (WT: 4.77 ± 0.43%ID/cc; *Slc6a8*^-/y^: 6.57 ± 0.78%ID/cc, *p* = 0.0007) (Fig. 6A). The difference in uptake can be detected in the pseudocolor images (brain is outlined by the red oval) that demonstrate increased activity in the *Slc6a8*^*-/y*^ mouse brain (B1, B2, B3 as individual mice) compared to WT (C1, C2, C3). Individual regions of the brain were quantified for [^18^F]FDG uptake as %ID/cc (Fig. 6D) : external capsule (WT: 4.82 ± 0.54; *Slc6a8*^*-/y*^: 6.90 ± 1.00, *p* = 0.0009), hypothalamus (WT: 3.83 ± 0.45; *Slc6a8*^*-/y*^: 5.73 ± 0.79, *p* = 0.0003), brain stem (WT: 4.53 ± 0.42; *Slc6a8*^*-/y*^: 6.19 ± 0.83, *p* = 0.0011), caudate and putamen (WT: 5.07 ± 0.58; *Slc6a8*^*-/y*^: 7.67 ± 1.15, *p* = 0.0005), central gray matter (WT: 4.79 ± 0.77; *Slc6a8*^*-/y*^: 6.44 ± 0.80, *p* = 0.0011), cerebellum (WT: 4.51 ± 0.56; *Slc6a8*^*-/y*^: 6.09 ± 0.80, *p* = 0.0012), fimbria (WT: 4.80 ± 0.60; *Slc6a8*^*-/y*^: 6.29 ± 0.87, *p* = 0.0030), globus pallidus (WT: 4.38 ± 0.51; *Slc6a8*^*-/y*^: 6.57 ± 1.14, *p* = 0.0015), hippocampus (WT: 4.57 ± 0.53; *Slc6a8*^*-/y*^: 6.24 ± 0.90, *p* = 0.0018), inferior colliculus (WT: 4.43 ± 0.65; *Slc6a8*^*-/y*^: 5.89 ± 0.84, *p* = 0.0029), internal capsule (WT: 4.53 ± 0.59; *Slc6a8*^*-/y*^: 6.41 ± 1.00, *p* = 0.0016), neocortex (WT: 4.81 ± 0.46; *Slc6a8*^*-/y*^: 6.80 ± 0.88, *p* = 0.0005), rest of midbrain (WT: 4.43 ± 0.60; *Slc6a8*^*-/y*^: 1.01 ± 1.00, *p* = 0.0055), superior colliculus (WT: 4.64 ± 0.70; *Slc6a8*^*-/y*^: 6.25 ± 0.89, *p* = 0.0022), thalamus (WT: 4.75 ± 0.64; *Slc6a8*^*-/y*^: 6.42 ± 1.18, *p* = 0.0087), amygdala (WT: 3.55 ± 0.48; *Slc6a8*^*-/y*^: 5.87 ± 0.83, *p* = 0.000096), anterior commissure (WT: 5.53 ± 0.74; *Slc6a8*^*-/y*^: 8.16 ± 1.29, *p* = 0.0009), and basal forebrain (WT: 4.59 ± 0.55; *Slc6a8*^*-/y*^: 7.05 ± 1.25, *p* = 0.0013). The only brain area that was without statistical significance between the WT and the CRT-1 mutant was the olfactory bulb: (WT: 6.79 ± 1.10; *Slc6a8*^*-/y*^: 7.76 ± 1.28, *p* = 0.1368).

To gain greater insight into the brain’s response to this increased metabolic activity based on glucose metabolism, we examined RNA expression of glycolysis-regulating proteins (Fig. 6E). Both *Slc6a8*^*-/y*^ (n = 7) and littermate control WT mice (n = 9) were studied and samples run in triplicate. GLUT1 and GLUT3, predominant glucose transporters in the brain, were examined for increased RNA expression in brain tissue of *Slc6a8*^*-/y*^ mice; no substantial change in expression of *Glut1* (1.12 ± 0.19-fold change) or *Glut3* (0.90 ± 0.13-fold change) compared to WT mice was found. Liver kinase B1 (LKB1) and calcium-calmodulin kinase kinase-2 (CaMKK2) are two upstream activators of AMP-activated protein kinase (AMPK), playing an important role in regulating cellular energy and responding to low cellular energy levels. Neither *Lkb1* (0.94 ± 0.13-fold change) nor *Camkkb* (0.97 ± 0.20-fold change) expression were upregulated compared to WT mice. Finally, we examined GAMT and AGAT, essential in the biosynthesis of endogenous cellular creatine. Neither *Gamt* (1.066 ± 0.11-fold change) nor *Agat* (0.95 ± 0.08-fold change) was increased compared to WT mice.

**Fig. 7:**
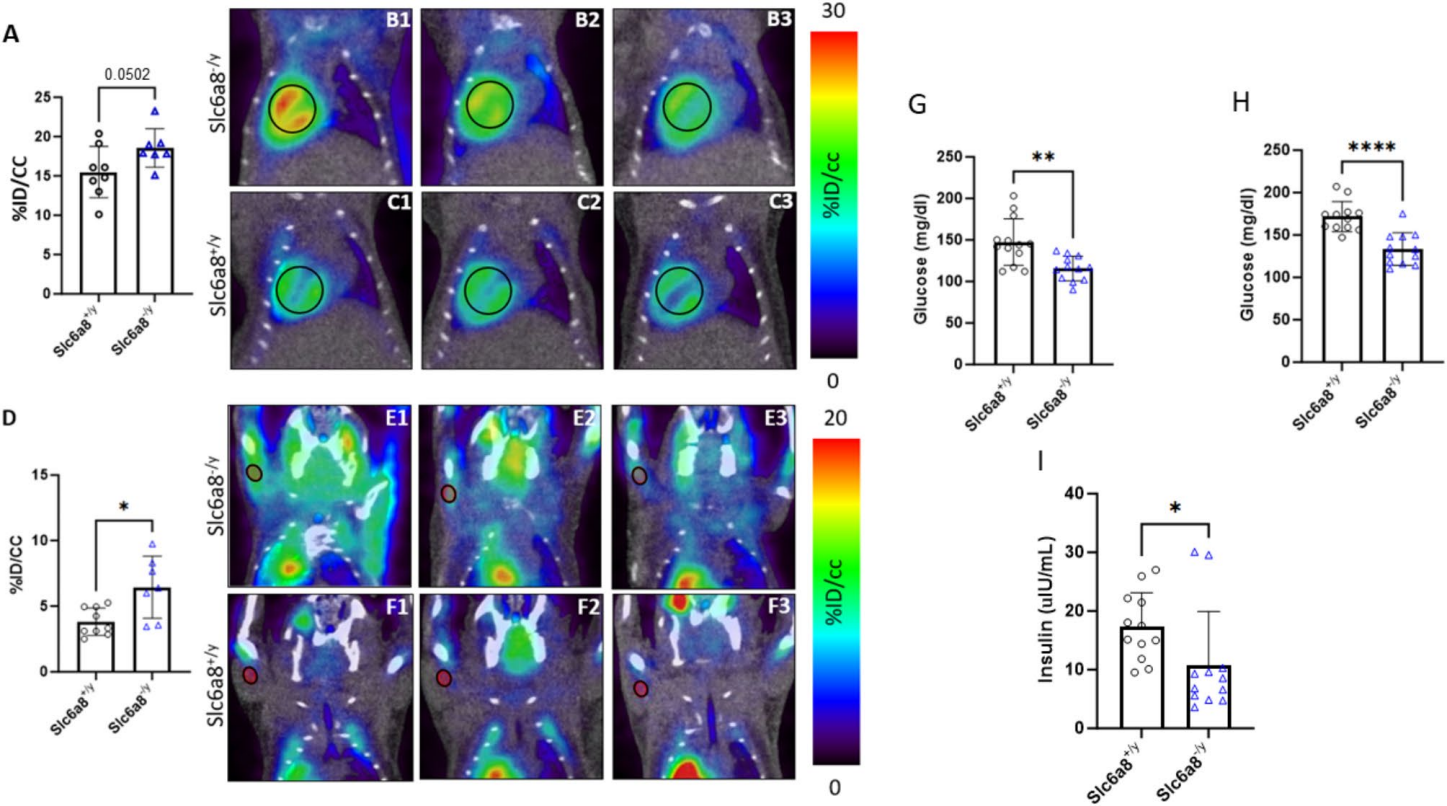
Increased glucose-mediated metabolic activity is detected in the heart and skeletal muscle. Glucose metabolic activity is increased in the heart of *Slc6a8*^*-/y*^ mice (**A**, representative images in **B1**, **B2**, **B3** [heart = circle]) compared to WT mice (representative images in **C1**, **C2**, **C3**). Similarly, glucose metabolic activity is increased in the skeletal muscle of *Slc6a8*^*-/y*^ mice (**D**, representative images in **E1**, **E2**, **E3**) compared to wild type mice (representative images in **F1**, **F2**, **F3**). Blood glucose is reduced in *Slc6a8*^*-/y*^ mice compared to wild type whether in the fed state (**G**) or fasted (**H**) Similarly, plasma insulin level is also decreased in *Slc6a8*^*-/y*^ mice (I). Data is presented as mean + standard deviation. *P* values of ≤ 0.05 are shown with one (*) asterisk, ≤ 0.01 are shown with two (**), and *P* values less than 0.0001 are shown with four (****).

Other organs/tissues also demonstrated increased glucose metabolic activity by [^18^F]FDG-PET (Fig. 7). The hearts of *Slc6a8*^*-/y*^ mice (Fig. 7A) demonstrated %ID/cc of 18.85 ± 2.76 [representative 3 mice in B1, B2, B3 (black circle = heart)] while hearts in WT mice averaged 15.68 ± 3.14%ID/cc (representative 3 mice in C1, C2, C3) (mean ± SD, n = 9 WT and n = 7 *Slc6a8*^*-/y*^; *p* = 0.0502). Analyzing the upper extremity (Fig. 7D), skeletal muscle also demonstrated differences based on creatine transporter activity (representative images in E1, E2, E3 [*Slc6a8*^*-/y*^] (black circle = skeletal muscle of upper extremity) and F1, F2, F3 [WT]) (WT: 3.81 ± 1.05%ID/cc [n = 9]; *Slc6a8*^*-/y*^: 6.45 ± 2.36%ID/cc [n = 7]; *p* = 0.0253).

To gain insight into the findings of Ensure preference testing and the increased glucose-mediated metabolic activity in both skeletal and cardiac myocytes, we measured whole blood glucose from both fed (Fig. 7G) and fasted mice (Fig. 7H) and insulin (Fig. 7I) from fasted mice. Glucose levels were reduced ~ 22% in *Slc6a8*^*-/y*^ mice compared to WT in both fed (WT [n = 12]: 147.3 ± 28.08 mg/dl vs. *Slc6a8*^*-/y*^ [n = 12]: 115.6 ± 14.78 mg/dl, *p* = 0.002 [21.5% reduction]) and fasted (WT [n = 12]: 171.6 ± 17.8 mg/dl vs. *Slc6a8*^*-/y*^ [n = 12]: 133.3 ± 19.09 mg/dl, *p* < 0.0001 [22.3% reduction]) states. Fasting plasma insulin levels were also reduced in *Slc6a8*^*-/y*^ mice (WT [n = 12]: 17.32 ± 5.80 mg/dl vs. *Slc6a8*^*-/y*^ [n = 12]: 10.80 ± 9.13 mg/dl, *p* = 0.0483). The reduced blood glucose and insulin levels are consistent with the increased glucose consumption in the brain, heart, and skeletal muscle.

### Serotonergic signaling is altered in the brain of the *Slc6a8*^-/y^ mouse

**Fig. 8:**
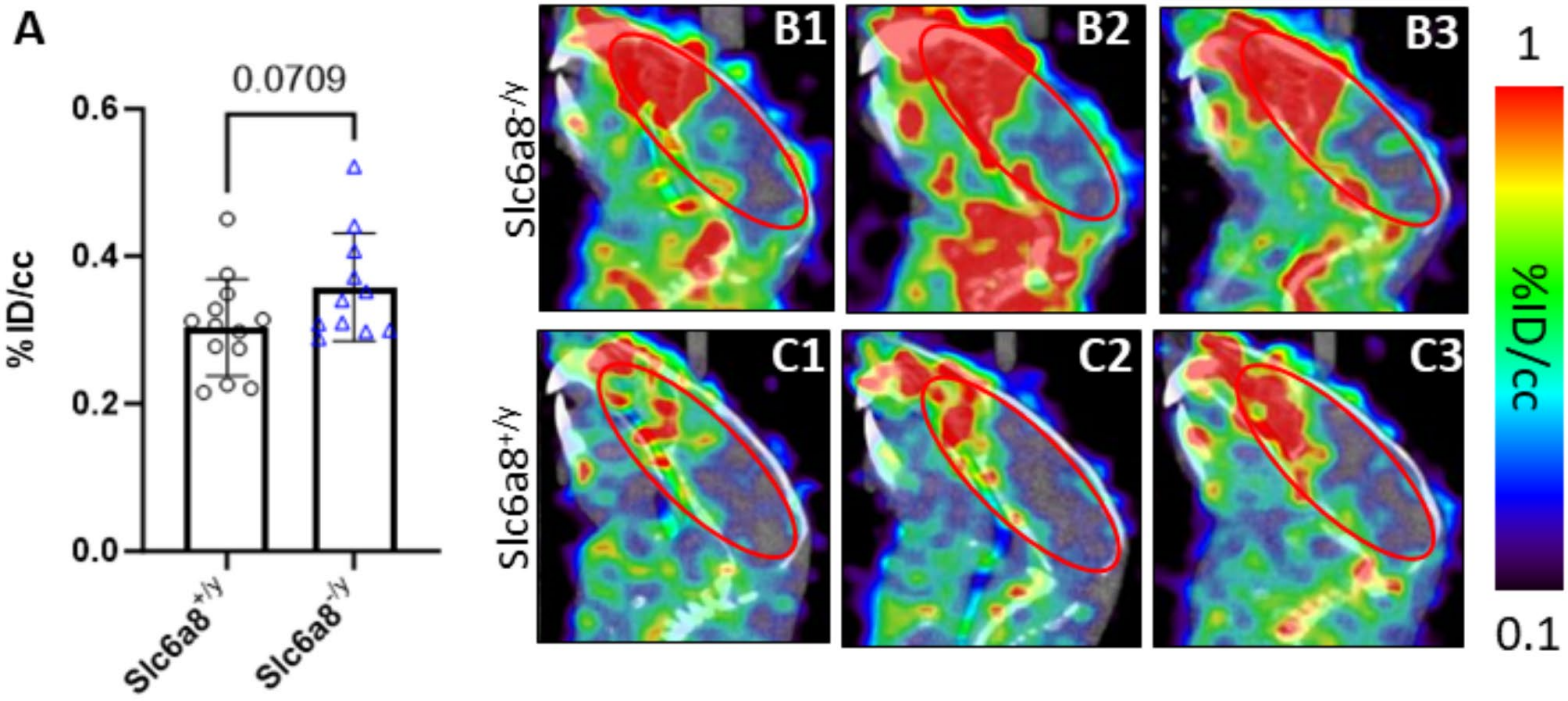
[^18^F]MPPF PET/CT imaging demonstrates serotonergic signaling abnormalities in* Slc6a8*^*-/y*^ mice. [^18^F]MPPF PET/CT data demonstrates increased uptake in *Slc6a8*^*-/y*^ mice (**A**, representative images in **B1**, **B2**, and **B3**) compared to WT (representative images in **C1**, **C2** and **C3**). Data is presented as mean + SD.

With the unique behavioral findings of lack of marble burying, reduced digging, and minimal nestlet shredding, along with the known involvement of serotonin in the conditioned fear stress-induced freezing behavior, we interrogated CNS serotonergic signaling with [^18^F]MPPF/PET (Fig. 8). Quantitative analysis of [^18^F]MPPF uptake in the brain was increased in mice lacking functional Slc6a8 with a strong trend towards statistical significance (*p* = 0.0709; WT, 0.304 ± 0.066%ID/cc, n = 13; *Slc6a8*^*-/y*^, 0.358 ± 0.073%ID/cc, n = 11) (Fig. 8A). Representative images of *Slc6a8* mutant mice demonstrate greater signal intensity in the brain (particularly the forebrain) from [^18^F]MPPF (Figure B1, B2, B3) than age-matched WT controls (Figures C1, C2, C3).

## Discussion

The creatine-phosphocreatine shuttle is an important system for the rapid regeneration of ATP in cells that have high energy demand such as the brain, heart, and skeletal muscle. In such highly active and energy consuming organs and tissues, phosphocreatine, generated by mitochondrial creatine kinase and ATP, acts as an energy reservoir. This grants the quick regeneration of cytoplasmic ATP from ADP by the reverse reaction of cytosolic creatine kinase; this allows for ATP to be available and levels to be kept stable for immediate energy needs. In this manner phosphocreatine acts as a mobile energy carrier, more diffusible than ATP, as the shuttle itself acts to store and release energy to prevent fluctuations in ATP levels. In the brain, the shuttle is essential as neurons require a constant supply of ATP for preservation of ion gradients, synaptic transmission and recycling of neurotransmitters amongst other activities necessary for brain development and function^6^. When CRT-1 transporter function is impaired, the phosphocreatine shuttle is defective and there is inadequate intracellular creatine thereby affecting the ability to regenerate ATP efficiently and leading to an energy deficit. This is particularly impactful on the brain as demonstrated in the marked behavioral abnormalities detected in CRT-1 mutated mice^19^ and in humans^3,51^ where cognitive impairment, developmental delay, absence of speech, seizures and autistic-like behaviors are present.

CRT-1 is the only known transporter of creatine in the brain, heart and skeletal muscle^52^. Thus, the knockout *Slc6a8*^*-/y*^ mice studied^19^ demonstrate markedly reduced plasma and brain creatine and creatinine. These studies determined that *Slc6a8*^*-/y*^ brain tissue creatine levels were ~ 21% of that found in wild type mice, similar to the ~ 31% found in another murine model of the disorder^53^. GAA, also transported by CRT-1^54^, is a key intermediate for CNS endogenous creatine synthesis, being transported from AGAT to GAMT expressing brain cells^54^. While CNS GAA accumulation is characteristic in GAMT deficiency^55^, it can also accumulate, albeit at substantially lesser levels, in the brains of patients with creatine transporter mutations^56^. Loss of function of Slc6a8 with intracellular trapping of GAA likely explains why plasma and CNS levels were higher in the *Slc6a8*^*-/y*^ mice compared to wild type controls.

The behavioral abnormalities in this mutated Slc6a8 mouse model were considerable: while abnormalities in fear conditioning response and novel object recognition (and including the Morris water maze), had been previously reported,^19^ anxiety-like behavior was conspicuous and new findings of alterations in open field, light dark transition, nestlet shredding, and marble burying were prominent; the latter two are considered natural or normal behaviors in mice and have been used in other settings to model anxiety disorders due to the excessive nature of this behavior^49^. In fact, the combination of altered marble burying and reduced nestlet shredding led us to suspect an alteration in serotonergic signaling in *Slc6a8* mutated mice: a previous study in wild type mice demonstrated that serotonin uptake inhibitors suppressed nestlet shredding and marble burying behavior^49^. The serotonergic (i.e., 5-hydroxytryptamine [5-HT]) system, as a CNS neurotransmitter and neuromodulator, contributes to the development of the brain, and in adults regulates a wide spectrum of brain physiology and behavior; in its impairment, a number of psychiatric disorders have been reported including anxiety, depression, schizophrenia, and autism^57–59^. 5-HT_1A_ receptors (mainly in raphe nuclei, and the hippocampus, cortex and limbic system) have been implicated in behavior and emotion and their modulatory role in anxiety-related behaviors is supported from studies with 5-HT_1A_ receptor knockout mice (exhibiting anxiety-like behavior)^60,61^ and transgenic mice overexpressing 5-HT_1A_ receptors (demonstrating decreased anxiety-like behavior)^62^. Furthermore, there is data suggesting that alterations in the serotonergic system impair fear behavior, specifically higher serotonin levels (such as by chronic selective serotonin reuptake inhibitor [SSRI] administration) in rodents, impairs acquisition of tone conditioning and fear learning and is correlated with impaired contextual auditory fear conditioning^48,63^. In the studies herein, [^18^F]MPPF is promptly taken up by the brain after administration and clears rapidly from the cerebellum but more slowly from target areas^64^; previous investigations have demonstrated that its distribution correlates with known human 5-HT_1A_ receptor localization^65,66^. Existing preclinical data has demonstrated that the binding of the radiopharmaceutical [^18^F]MPPF reflects the density of 5-HT_1A_ receptors (but not necessarily the functional state due to potential internalization^67^).

In these studies, conducted with Slc6a8 mutated mice utilizing [^18^F]MPPF, we were able to detect by PET/CT imaging supportive evidence (*p* = 0.0709) of increased 5-HT_1A_ receptor binding in the brains of Slc6a8 mutated mice. This finding, and the role of serotonergic signaling, suggest an underlying mechanism for the anxiolytic, anti-obsessive compulsive-like behavior found in these studies with *Slc6a8*^*-/y*^ mice^68^. There is also existing and supporting biochemical preclinical data of alterations in serotonergic signaling in these mice^19,69^. *Slc6a8*^*-/y*^ mice were found to have increased brain (i.e., prefrontal cortex, hippocampus, neostriatum) 5-hydroxyindoleacetic acid (5-HIAA, the primary metabolite of 5-HT), increased hippocampal monoamine oxidase levels (MAO, an enzyme metabolizing 5-HT), and increased hippocampal tryptophan hydroxylase-2 (TPH-2, an enzyme in the biosynthetic pathway of serotonin), all related to serotonin metabolism and synthesis^68^.

**Fig. 9:**
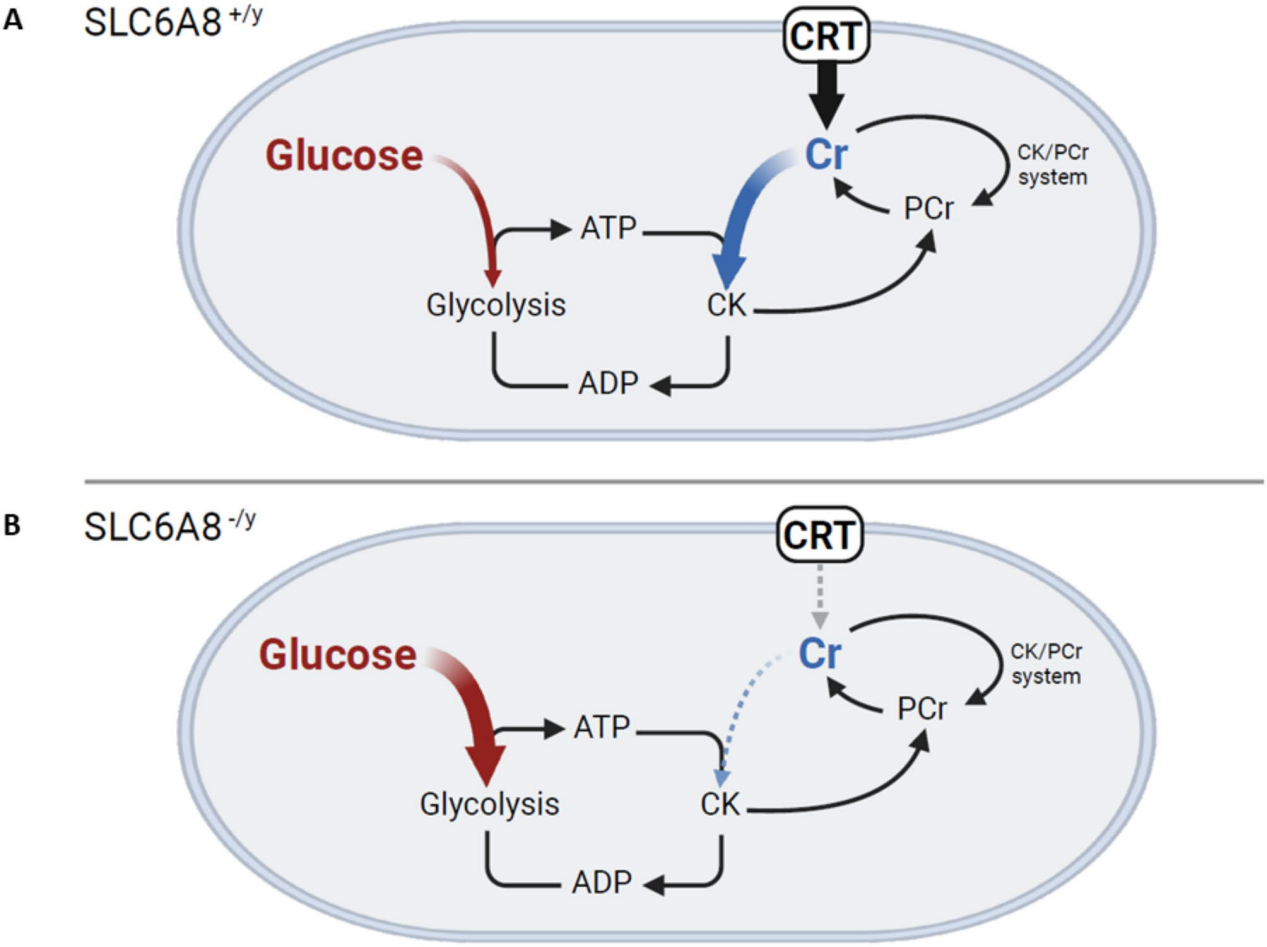
A model of the effect of loss of function of the creatine transporter on glucose uptake. The normal creatine/phosphocreatine shuttle is represented in (**A**) while (**B**) is a model of what occurs when the creatine transporter is mutated and the brain relies on glucose solely as the source of energy. (ATP, adenosine triphosphate; ADP, adenosine diphosphate; CRT, creatine transporter; Cr, creatine; PCr, phosphocreatine; CK, creatine kinase.

The brain relies substantially on both creatine (from endogenous production and Slc6a8-mediated transmembrane transport) and glucose for energy production (Fig. 9A). With a reduction in cellular creatine through loss of the functional creatine transporter, the creatine-phosphocreatine shuttle is altered, forcing the brain to rely further on immediate glucose metabolism for ATP production than stored energy as creatine phosphate (Fig. 9B). By use of [^18^F]FDG-PET imaging, this increase in glucose metabolism in the brain of CRT-1 mutated mice was revealed. Anatomic analysis of brain regions demonstrated that CRT-1 mutant mice had highly statistically significant increases in glucose-mediated metabolic activity in all areas except for the olfactory bulbs; while sensory processing issues (e.g., hearing, visual) may be anecdotally reported in afflicted CRT-1 mutated patients, alteration in olfaction is not a common finding. As a consequence of the reduced availability of creatine to serve as an energy reservoir, the *Slc6a8*^*-/y*^ brain, regardless of the shift to utilize more glucose, has reduced capability for buffering ATP levels and meeting the immediate energy needs to support its normal function.

Very recently another group also examined [^18^F]FDG-PET as a biomarker for brain metabolic activity in *Slc6a8*^*-/y*^ mice^70^. While they concluded lower uptake of [^18^F]FDG in the brains of *Slc6a8*^*-/y*^ mice, their experimental and quantification methods were different and this may at least partially explain the discrepancy between their conclusions with ours. This includes differences in age of the mice, mouse weights, method of injection of [^18^F]FDG (intraperitoneal in their case, intravenous in ours), conscious status of mice at time of [^18^F]FDG administration (awake injection with potential for stress-related effects on brain glucose uptake in their case, unconscious injection in our case), differences in time for [^18^F]FDG uptake (45 min in their case, 1 h in ours), and, importantly, the method of PET data quantification. In their case, a semiquantitative measurement of radiotracer that was taken up by the brain as standardized uptake value ratio [SUVR] calculated by dividing the tissue concentration of the tracer by the injected dose, normalized to the weight and further divided by the value obtained specifically from the brainstem. While the SUVR method is used most commonly in the clinical field^71^, the selection of the brainstem region for normalization may affect data interpretation. We, instead, utilized a more traditional approach, and what is usually reported for preclinical validation, as % injected dose per cubic centimeter, where a density of 1 g/cubic centimeter in tissue is assumed. In addition, our data demonstrated differences in [^18^F]FDG PET signal between the groups at the brainstem, suggesting to us that normalizing the PET data to values from this region by Disdier^70^ is not appropriate.

The increased need for glucose in the brain (and other organs, in particular skeletal muscle^53^ and heart) may explain why whole blood glucose levels are reduced (whether fasted or fed), why *Slc6a8*^*-/y*^ mice consume more Ensure (2.5 g of sugar per ounce) than do wild type mice, as well as the insulin level differences when compared with wild type controls. However, there is an alternative explanation: alterations in the serotonergic system result in increased sucrose consumption in *Slc6a8*^*-/y*^ mice and lower blood glucose than wild type mice. Male and female 5-HT_1B_^-/-^ and female 5-HT_1A_^-/-^ mice have been found to drink more sucrose-containing water than WT mice and male 5-HT_1B_^-/-^ mice have a lower fasting glucose^72^. 5-HT_1B_ receptors (primarily presynaptic, especially in the striatum, basal ganglia, and cortex) have a role in ingestive behavior^73^, providing further evidence of alterations in the serotonergic system to the findings demonstrated in this present study.

While there is increased metabolic activity and reliance on glucose for ATP production in the brain of creatine transporter mutated mice, there appear to be limitations as to how cells may respond to decreased energy charge in the CNS. There are a number of glucose transporters in the brain, with, in mice and humans, two being predominant: GLUT1, responsible for transporting glucose across the BBB and ensuring a steady supply of glucose (insulin-insensitive and abundant in astrocytes and brain vasculature) and GLUT3 (predominant in neurons)^74^, with a higher affinity for glucose to meet neuronal high energy demands. These are known to increase in the postnatal period^75^. However, neither Glut1 nor Glut3 RNA expression was found to be increased in the CRT-1 mutated mouse brain in comparison with controls. This is unlike Glut4 which is increased in the skeletal muscle in *Slc6a8*^*-/y*^ mice^53^.

Other mechanisms of response to reduced cellular energy may also be limited. AMP-activated protein kinase (AMPK) is the main cellular protein acting as a sensor of energy charge, regulating processes that consume or regenerate ATP, and signaling when a cell has low energy levels, being activated by increased cellular AMP^76,77^. Liver kinase B1 (Lkb1), expressed in the brain and concentrated in the cell nucleus, encodes a kinase that directly phosphorylates, thus activating, AMPK: Lkb1 was not increased in the brain of *Slc6a8*^*-/y*^ mutated mice. Calcium/calmodulin dependent protein kinase (CaMKKB), a major kinase highly abundant in the brain^78^ regulating calcium-mediated activation of AMPK, was also not increased in *Slc6a8*^*-/y*^ mutated mice compared to wild type controls. Examining RNA expression of Gamt and Agat, both critical to the synthesis of creatine, demonstrated no increase in the brains of *Slc6a8*^*-/y*^ mutated mice. In aggregate, these findings suggest that in the case of low energy conditions as with mutated Slc6a8, the brain may be challenged to upregulate enzymes or transporter number to facilitate the rapid regeneration of ATP. This may clarify why even with increased glucose metabolic activity in the brain, functional deficits remain as demand outpaces energy supply from glucose alone.

While the hallmark of creatine transporter deficiency is neurological with developmental abnormalities, the effect of loss of creatine transporter activity in other highly energy dependent organs and tissues was also found as reflected in increased glucose metabolic activity. Cardiomyopathy, heart failure and arrhythmias have been found in creatine transporter patients and mice^79^. [^18^F]FDG PET/CT imaging of the *Slc6a8*^*-/y*^ mouse demonstrated a strong statistical trend (*p* = 0.0502) towards increased glucose-mediated metabolic activity in the heart. In addition, *Slc6a8*^*-/y*^ mice demonstrated statistically significant increased glucose-mediated metabolic activity in skeletal muscle.

## Conclusions

The preclinical data herein support the potential use of [^18^F]FDG in loss of function mutations of SLC6A8 to detect changes in brain metabolism in assessing preclinical models and possibly patients as new therapies are developed. It also supports the use of [^18^F]MPPF to explore and assess changes in the serotonergic signaling pathways as an area of dysfunction that may be amenable to existing pharmacologic interventions. These diagnostic agents, aimed at localization and detection of alteration in function, may have potential beyond assessment of metabolic activity but also in our further understanding of the underlying physiologic processes and dysfunction that occur in this disorder of creatine uptake and cellular metabolism.

## Data Availability

The data that support the findings of this study are available from the corresponding author upon reasonable request.
